# Defining the kinetics of severe fever with thrombocytopenia syndrome virus acquisition and dissemination in naturally-infected *Haemaphysalis longicornis*

**DOI:** 10.3389/fcimb.2025.1706970

**Published:** 2025-11-03

**Authors:** Eliane Esteves, Clemence Obellianne, Ahmed Garba, Shovon Lal Sarkar, Margaret G. Schuler, Meghan E. Hermance

**Affiliations:** Department of Microbiology and Immunology, Frederick P. Whiddon College of Medicine, University of South Alabama, Mobile, AL, United States

**Keywords:** *Haemaphysalis longicornis*, severe fever with thrombocytopenia syndrome virus, virus-tick interactions, tick-borne virus, transstadial transmission, virus dissemination within tick, *Dabie bandavirus*

## Abstract

*Haemaphysalis longicornis* is a primary vector of severe fever with thrombocytopenia syndrome virus (SFTSV), an emerging virus of public health concern that can cause severe disease and high mortality rates. For zoonotic tick-borne viruses such as SFTSV, it is critical that specific tick-virus pairings are carefully examined to elucidate the intra-tick infection dynamics that enable viral infection, dissemination, and persistence within a particular tick species. This study investigated the intra-tick kinetics of SFTSV acquisition and dissemination in *H. longicornis* by feeding nymphs on viremic mice. Nymphs were collected and processed at defined time points during and after feeding, as well as post-molting. Viral RNA was detected in nymph bodies within the first 24 hours of feeding, and infectious virus was subsequently detected at 48 hours. The rates of SFTSV acquisition by *H. longicornis* nymphs were consistently high across all time points. For infected ticks to be capable of transmitting virus during a subsequent blood meal, the virus must disseminate beyond the tick midgut and ultimately infect the salivary glands. Thus, the kinetics of virus dissemination beyond the midgut and into the hemolymph were evaluated by screening nymph legs for the presence of virus. SFTSV was capable of early dissemination beyond the nymph midgut during blood feeding, as well as at time points after the nymphal blood meal was complete. Furthermore, SFTSV RNA was detected in various tissues of molted adults that had acquired virus as nymphs, and these results demonstrated that time post-molting influences the efficiency and level of virus maintained by transstadial transmission. This is the first study using naturally-infected ticks to demonstrate the kinetics of viral dissemination beyond the midgut for any tick-borne virus. These findings offer new insights into tick-virus interactions that can ultimately guide strategies aimed at disrupting virus maintenance and transmission by ticks.

## 1 Introduction

Ticks are obligate hematophagous ectoparasites that efficiently transmit viruses, bacteria, and protozoa through their blood-feeding behavior (Jongejan and Uilenberg, 2004; Dantas-Torres et al., 2012; Otranto et al., 2013). These pathogens are responsible for numerous tick-borne diseases of both medical and veterinary importance, including but not limited to Lyme disease, babesiosis, anaplasmosis, theileriosis, ehrlichiosis, and viral diseases (Madison-Antenucci et al., 2020). Among tick-borne viral diseases, Severe Fever with Thrombocytopenia Syndrome (SFTS), caused by the Severe Fever with Thrombocytopenia Syndrome Virus (SFTSV), recently emerged as a major public health concern and has been included in the World Health Organization’s list of priority pathogens (World Health Organization, 2024). SFTSV, recently renamed *Bandavirus dabieense* (International on Committee of Taxonomy Viruses: ICTV, 2024), belongs to the genus *Bandavirus*, within the family *Phenuiviridae* and the order *Hareavirales*. Following the initial isolation of SFTSV from a patient in China in 2009 (Yu et al., 2011), numerous additional human cases have been reported across several countries in East and Southeast Asia (Yu et al., 2011; Takahashi et al., 2014; Kim et al., 2018; Tran et al., 2019; Ongkittikul S WRaRP, 2020; Peng et al., 2020; Win et al., 2020; Zohaib et al., 2020). Clinical symptoms of SFTSV include acute febrile illness characterized by high fever, thrombocytopenia, leukopenia, elevated liver enzymes, and gastrointestinal symptoms such as nausea, vomiting, and diarrhea. In severe cases, the disease may progress to multi-organ dysfunction, hemorrhagic manifestations, and central nervous system involvement, particularly in elderly or immunocompromised individuals (Yu et al., 2011; Sun et al., 2014; Seo et al., 2021; Li et al., 2022). The overall case fatality rate of SFTS ranges from approximately 5% to 30%, with variations influenced by several key factors, including patient-related characteristics such as advanced age and immunocompromised status, as well as viral factors such as circulating genotype of SFTSV (Casel et al., 2021).

SFTSV can be transmitted via direct contact of infected body fluids between humans (Wu et al., 2022; Zhang et al., 2024). Zoonotic transmission has also been demonstrated, particularly through close contact with infected domestic animals such as cats and dogs (Yamanaka et al., 2020; Miyauchi et al., 2022). However, SFTSV is mainly considered to be a tick-transmitted pathogen, and extensive field studies have detected the virus in questing and host-attached ticks across SFTS endemic regions (Wang et al., 2015; Sato et al., 2021). Although SFTSV has been detected in multiple tick species, including *Haemaphysalis longicornis, Haemaphysalis flava*, *Amblyomma testudinarium*, *Ixodes nipponensis*, *Rhipicephalus microplus*, and *Dermacentor silvarum* (Yun et al., 2014; Jo et al., 2019; Hu et al., 2020), *H. longicornis* is considered the primary vector for SFTSV. Laboratory vector competence studies demonstrated the ability of *H. longicornis* to acquire, maintain, and transmit SFTSV (Luo et al., 2015; Zhuang et al., 2018; Zhao et al., 2022). Other tick species, including *H. flava* and *Ixodes sinensis*, have also been demonstrated as competent vector species for SFTSV (Hu et al., 2020; Fang et al., 2023); however, *H. longicornis* is considered the primary vector for the virus based on strong correlations between the presence of this tick species in nature and SFTS disease incidence (Liu et al., 2015).

*Haemaphysalis longicornis*, commonly known as the Asian longhorned tick, is native to East Asia, particularly China, Japan, Korea, and parts of eastern Russia (Zhao et al., 2020; Yabsley and Thompson, 2023). Over time, *H. longicornis* spread beyond its original range and also established in New Zealand and parts of Australia in the early 20th century (Heath, 2016). In addition to its presence in the Asia-Pacific region, *H. longicornis* populations recently established in the Western Hemisphere (Bajwa et al., 2024) and have since been documented in at least 20 states across the eastern United States (Myers and Scimeca, 2024; U.S. Department of Agriculture, Animal and Plant Health Inspection Service, 2024a). Specific biological characteristics exhibited by *H. longicornis* likely contribute to its capacity to establish in a wide range of ecological environments. Among these are its different reproductive strategies, its ability to parasitize a wide range of vertebrate hosts, and its ecological adaptability. Notably, *H. longicornis* has the capacity for parthenogenesis and bisexual reproduction. Through parthenogenesis, females can produce offspring without mating, allowing a single female to establish a new population. This reproductive strategy is especially advantageous for the species colonization in new areas, as it eliminates the dependence on males. In contrast, bisexual reproduction contributes to genetic diversity, which may enhance the species’ adaptability in established populations (Harry Hoogstraal et al., 1968; Chen et al., 2012; Zhao et al., 2024). In addition to its reproductive flexibility, *H. longicornis* is a host generalist, capable of parasitizing a broad range of vertebrate hosts, including wild and domesticated animals (Heath, 2016; Tufts et al., 2021; Thompson et al., 2022; U.S. Department of Agriculture, Animal and Plant Health Inspection Service, 2024b). The remarkable ecological adaptability exhibited by *H. longicornis*, particularly its broad tolerance to climatic variability, enables it to survive across a wide temperature range and thrive in diverse environmental conditions, from temperate to subtropical regions (Heath, 2016; Rochlin, 2019). These traits have enabled the tick’s successful colonization of new geographic areas and contribute significantly to its invasive potential (Heath, 2016; Rochlin, 2019).

Our previous work demonstrated that *H. longicornis* is capable of transovarial transmission of the North American tick-borne bandavirus, Heartland virus (HRTV) (Raney et al., 2022b), which is closely related to SFTSV and is endemic to several of the U.S. states that *H. longicornis* recently established invasive populations within. In these experiments, mice exposed to the HRTV-infected *H. longicornis* seroconverted relative to HRTV, suggesting that *H. longicornis* are also capable of horizontal (tick-to-host) transmission of the virus (Raney et al., 2022b). Given the recent trans-hemispheric dispersion of *H. longicornis*, the increasing incidence of SFTS human cases, and the possibility that invasive populations of *H. longicornis* could intensify transmission of endemic tick-borne bandaviruses such as HRTV, *H. longicornis* and tick-borne bandaviruses pose an increasing public health threat. Therefore, it is critical to fill fundamental gaps in knowledge related to intra-tick infection and dissemination dynamics that enable viruses such as SFTSV to be maintained and transmitted by *H. longicornis*.

To be transmitted by competent ixodid tick vectors, tick-borne viruses must be capable of surviving the tick’s unique physiological processes, including the multi-day feeding event that occurs only once per larval, nymphal, and adult life cycle stage, intracellular bloodmeal digestion, and the molting process where a tick transitions from one life cycle stage to the next. Uninfected ticks can initially acquire virus through blood feeding, either by taking a bloodmeal from a viremic vertebrate host or by feeding in close proximity to an infected co-feeding tick (Nuttall et al., 1994). As the tick feeds and acquires virus from the host, the first tick organ system that the virus contacts is the alimentary canal (Nuttall et al., 1994; Nuttall, 2013).

In ticks that are competent vectors, the virus is able to disseminate beyond the tick midgut and into the hemocoel (Nuttall, 2013). Little is known about this process, including whether virus escapes the midgut prior to molting, or whether virus escapes the midgut during molting and disseminates to distal tissues amidst the tissue re-organization associated with molting. In competent tick vectors, virus ultimately reaches the salivary glands and is subsequently capable of being transmitted in the saliva during the tick’s next blood meal. Since ixodid ticks feed once per larval, nymphal, and adult life cycle stage, ticks that acquire virus during blood feeding are then only able to horizontally transmit the virus to a vertebrate host after they molt to the next life cycle stage and take a subsequent blood meal (Nuttall, 2013). Therefore, the virus must be capable of surviving the tick’s molting event in what is known as transstadial survival.

Few studies have examined the intra-tick dynamics of virus infection, dissemination, and transstadial maintenance. *Hyalomma* species ticks are the major vector for Crimean-Congo hemorrhagic fever virus (CCHFV; family: *Nairoviridae*; order: *Hareavirales*), which is a segmented, negative-strand RNA virus in the same order (*Hareavirales*) as SFTSV. One study assessed *Hyalomma marginatum* acquisition and dissemination of CCHFV by feeding adult ticks on viremic STAT-1 knockout mice. After six days of feeding, the ticks were removed, their organs dissected, and all dissected organs from every tick screened positive for viral RNA (Gargili et al., 2013). This study also provided some initial insights into the transstadial transmission of CCHFV as nymphal *H. marginatum* were infested on the viremic STAT-1 mice, then viral RNA was detected in dissected tick salivary glands after the nymphs molted to adults (Gargili et al., 2013). In a separate study, the effects of time and blood feeding on CCHFV replication and dissemination were evaluated in *Hyalomma truncatum* adults intracoelomically injected with CCHFV (Dickson and Turell, 1992). Higher viral titers and infection rates were detected in reproductive tissues and salivary glands of fed ticks compared to unfed ticks, while CCHFV titers remained the same in other tissues regardless of tick feeding status (Dickson and Turell, 1992). These findings suggest that tick attachment and feeding stimulates viral replication in certain tick tissues; however, the intracoelomic injection of CCHFV bypassed the requirement for virus to escape the tick midgut, thereby providing unimpeded access for the virus to infect other tick tissues. This highlights the importance of utilizing ticks that are naturally-infected via blood-feeding on viremic animals in studies that characterize the intra-tick kinetics of virus acquisition, as well as virus dissemination beyond the tick midgut.

Given the many unknowns of the intra-tick viral infection and maintenance dynamics, it is clear that specific tick-virus interactions should be examined temporally in order to fully understand the kinetics of virus dissemination beyond the midgut relative to the molting event. When parthenogenetic lineages of *H. longicornis* nymphs are infested on small laboratory animals such as mice and fed until repletion, they feed for an average of five days before detaching (Zhao et al., 2024; Esteves et al., 2025). These *H. longicornis* nymphs then complete their molting process and emerge as adults within approximately three and a half weeks in laboratory settings. Although the life-stage specific timelines of feeding and molting are well established for many tick species, the timeline of tick molting relative to virus dissemination beyond the midgut is unknown for all tick-borne viruses, including SFTSV. Therefore, the present study applied a systematic time course to address the enduring gaps in knowledge of the intra-tick virus infection and dissemination kinetics. To determine the kinetics of SFTSV acquisition by and dissemination within *H. longicornis*, naïve nymphs were fed upon viremic mice, and nymphs were then collected and processed at defined time points during and after feeding on SFTSV-infected mice.

## 2 Materials and methods

### 2.1 Ethics and biosafety

All procedures involving uninfected ticks and vertebrate animals were carried out in Animal Biosafety Level 2 (ABSL-2) facilities. SFTSV-infected ticks were maintained in Arthropod Containment Level 3 (ACL-3) facilities, and experiments with SFTSV-infected animals and SFTSV-infected ticks were carried out in Animal Biosafety Level 3 (ABSL-3) facilities. All biosafety and vertebrate animal procedures were performed in accordance with protocols approved by the Institutional Biosafety Committee (protocol # 1619205) and the Institutional Animal Care and Use Committee (protocol # 2064154 and protocol # 2123859).

### 2.2 Mice and ticks

Five-week-old male BALB/c mice were purchased from the Jackson Laboratory (Bar Harbor, ME) and acclimated to the environment for one week prior to the start of an experiment. A pathogen-free *H. longicornis* colony, originated from parthenogenetic ticks collected in New York state, was procured through BEI Resources in 2020 and has since been maintained under laboratory conditions at the University of South Alabama. Off-host tick life cycle stages were kept in a desiccator at 21 °C with 95–99% relative humidity (RH), under a 16:8 light:dark photoperiod. The pathogen-free tick colony was maintained by feeding on Hartley guinea pigs and mice in ABSL-2 facilities. Nymphal *H. longicornis* included in Experiments I and II (described below) were fed as larvae on guinea pigs, whereas nymphs included in Experiment III were fed as larvae on mice. For use in the *in vivo* experiments described below, nymphs included in Experiments I and III were 2.5 months post-molt, whereas nymphs included in Experiment II were 4 months post-molting.

### 2.3 Cells and virus

African green monkey kidney (Vero E6) cells were cultured in Dulbecco’s Modified Eagle Medium (DMEM) with 10% fetal bovine serum (FBS) and 1% penicillin-streptomycin and maintained at 37 °C with 5% CO_2_. The YL1 strain of SFTSV was obtained from the World Reference Center for Emerging Viruses and Arboviruses (WRCEVA) at the University of Texas Medical Branch. The virus stock had previously been passaged twice on Vero cells and once in suckling mouse brain. Upon receipt, it was then passed twice in Vero E6 cells, and the viral titer was determined by focus forming assay (FFA), as previously described (Esterly et al., 2025).

### 2.4 Experimental design

**Figure 1:**
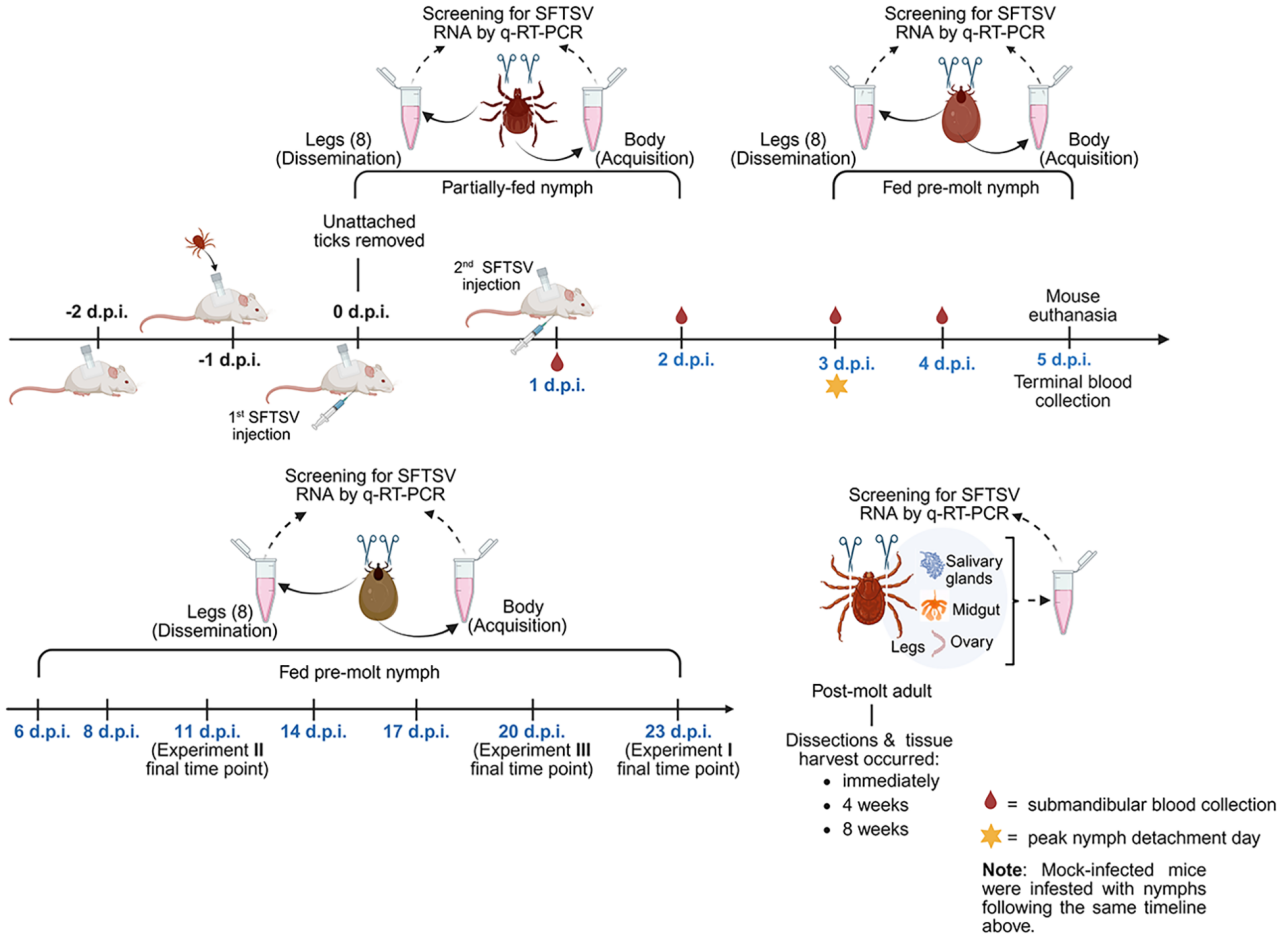
Overview of *in vivo* experiment timeline and major activities. Three independent *in vivo* experiments were performed to assess the kinetics of SFTSV acquisition and dissemination in *Haemaphysalis longicornis* nymphs fed on SFTSV-infected mice. The diagram outlines the timeline of tick infestation, mouse SFTSV injections, tick feeding and detachment, mouse blood collection and euthanasia, and tick collection time points. All time points are relevant to 0 days post-infection (d.p.i.), which was the first day mice were injected with virus. Partially-fed and fed pre-molt nymphs were collected at time points ranging from 1–23 d.p.i., shown in blue font. Upon collection, each nymphal tick was washed and legs were carefully severed. The body and corresponding legs from each nymph were labeled and processed separately for detection of viral RNA by q-RT-PCR. Nymphs that molted to adults were dissected at 3 time points post-molt. Dissected adult tick organs were also screened for viral RNA. Created by Biorender.com (https://BioRender.com/qi4bzqk).

To examine the acquisition and dissemination kinetics of SFTSV by *H. longicornis* nymphs, three independent *in vivo* experiments were performed under controlled laboratory conditions. Each experiment was designed to assess virus acquisition by *H. longicornis* nymphs feeding on viremic mice, as well as subsequent dissemination of virus beyond the nymph midgut at different developmental stages: partially-fed nymphs, fed pre-molt nymphs, and post-molt adults (**Figure 1**). Tick capsules were affixed to the dorsum of each mouse as previously described (Esteves et al., 2025). The next day, pathogen-free *H. longicornis* nymphs were transferred into the capsules. An average of 25–30 nymphs were infested per mouse in Experiments I and III, while 12–14 nymphs were infested per mouse in Experiment II. Twenty-four hours after the tick infestation, unattached nymphs were removed from the capsules and mice were randomly assigned to either the SFTSV-infected or the mock-infected group. Mice were intraperitoneally injected with the first dose of SFTSV (1.46 x 10^7^ focus-forming units [FFU] in 1.0 mL), or with an equivalent volume of DMEM media, at 0 days post-infection (d.p.i.). At 1 d.p.i., mice were injected with a second dose of SFTSV (3.65 x 10^6^ FFU in 0.25 mL), or with an equivalent volume of DMEM. The following numbers of mice were included in the SFTSV-infected and mock-infected cohorts for each of the three experiments: eight SFTSV-infected mice and two mock-infected mice for Experiments I and II; 10 SFTSV-infected and three mock-infected mice for Experiment III. At designated time points, partially-fed nymphs and fed pre-molt nymphs were collected and processed as described below. Mice were maintained until 5 d.p.i., at which point all nymphs had naturally detached and mice were euthanized.

#### 2.4.1 Daily mouse observations, blood collection, and necropsy

All procedures involving mouse manipulations, including tick capsule fixation and repair, tick transfer and collection, and submandibular blood sampling, were performed under isoflurane anesthesia (Esteves et al., 2025). For Experiments I and II, submandibular blood was collected from each mouse every other day from 1–4 d.p.i., whereas all mice in Experiment III were bled daily from 1–4 d.p.i. Submandibular blood samples were stored in microtubes containing 500 µL of TRIzol reagent (Life Technologies) for future RNA extraction. Mice were monitored daily for clinical signs of disease, including weight loss, changes in appearance (e.g., reduced grooming, ruffled coat, ocular or nasal discharge, hunched posture), respiratory distress, neurological symptoms, and abnormal behavior. Mice exhibiting any of the following humane endpoints were immediately euthanized: greater than 20% body weight loss, paralysis, seizures, gasping respiration, hemorrhage, prostration, or unresponsiveness. Euthanasia was performed by isoflurane overdose, followed by terminal cardiac puncture and cervical dislocation. At 5 d.p.i. (**Figure 1**), surviving mice from all experiments were euthanized, and terminal blood was collected via cardiac puncture. Terminal blood samples were transferred into microtubes containing 500 µL of TRIzol reagent for future RNA extraction.

#### 2.4.2 Nymph collection and sample preparation

Partially-fed and fed pre-molt *H. longicornis* nymphs were collected at designated time points ranging from 1–23 d.p.i. in Experiments I–III (**Figure 1**). In Experiment I and III, upon collection, intact nymphs were washed in 70% ethanol, air-dried and transferred to a petri dish. The eight legs of each nymph were then carefully separated from the body using a scalpel under dissecting microscope. Care was taken to manipulate the nymphs gently during leg severing, as too much pressure applied to the nymph body can result in the midgut contaminating tick leg samples, which is apparent when the hemolymph exudate from the severed legs becomes discolored. Any leg samples collected with midgut contamination were discarded and the corresponding tick body sample was also removed from the study. To ensure no sample cross-contamination, a new scalpel blade and petri dish work surface was used for each tick. Each set of nymph legs and the corresponding nymph body were transferred into a 2 mL reinforced polypropylene microtube containing 500 µL of TRIzol reagent. Nymph leg and body samples from Experiments I and III were homogenized directly in TRIzol for future RNA extraction. A combination of three 3-mm beads and four 2-mm sterile stainless-steel beads were used to homogenize tick leg samples on a Tissue Lyser II (Qiagen) at 30 Hz for 3 minutes, while only three 3-mm sterile stainless-steel beads were added to the tube to homogenize nymph body samples. In Experiment II, the surface of each nymph was decontaminated by sequentially washing in 10% bleach, 70% ethanol, and Milli-Q water. After surface decontamination of Experiment II nymphs, legs were severed as described above, and each set of nymph legs and the corresponding nymph body were transferred into a 2 mL reinforced polypropylene microtube containing 600 µL of DMEM (supplemented with 2% FBS, 1% penicillin-streptomycin, and 2.5 ng/mL amphotericin B). The stainless-steel beads and Tissue Lyser II were applied to the samples as described above. Experiment II samples were then briefly centrifuged, and a 200 µL fraction of clarified homogenate was transferred to microtubes containing 600 µL of TRIzol reagent for future RNA extraction while the remaining 400 µL fraction was stored at −80 °C until use in infectious virus assays.

#### 2.4.3 Adult tick dissections, tissue collection, and sample preparation

After molting from nymphs, adult *H. longicornis* were dissected at the following time points: within one week post-molt (corresponding to immediately pos-molt), four weeks post-molt, and eight weeks post-molt (**Figure 1**). Adult ticks were washed with 70% ethanol, air-dried, and their legs were removed and processed using the same procedure described above for the nymphs. To collect the organs of adult ticks, each tick was immersed in a drop of phosphate-buffered saline (PBS) on a petri dish under a dissecting microscope. Dissection was performed using a scalpel, starting with an incision at the base of the capitulum and continuing along the edge of the tick’s body to open the dorsal cuticle and expose internal organs. Salivary glands, midgut, and ovaries were carefully removed and washed twice in fresh PBS, then transferred into microtubes containing 100 µL of TRIzol reagent and kept on ice. To prevent cross-contamination, a new scalpel blade and petri dish were used for each tick. The dissected tissues were homogenized in TRIzol using a pellet pestle mixer (Thermo Fisher Scientific). After homogenization, an additional 400 µL of TRIzol reagent was added to each sample and stored until RNA extraction.

### 2.5 RNA extraction and detection of viral RNA by q-RT-PCR

A hybrid RNA extraction protocol combining TRIzol reagent and the RNeasy Mini Kit (Qiagen) was used to extract RNA from tick and mouse-derived samples, as previously described (Hermance and Thangamani, 2015; Raney et al., 2022a). RNA concentrations were measured using a NanoDrop One spectrophotometer (Thermo Fisher Scientific). Viral RNA levels of SFTSV were determined by absolute quantification using quantitative reverse transcription real-time PCR (q-RT-PCR). RNA extracted from tick samples, including bodies, legs, and dissected tissues, as well as from mouse blood, was used as a template for one-step q-RT-PCR reactions. Amplification was performed using the LightCycler 480 II PCR System (Roche) with the iTaq Universal Probes One-Step Kit (Bio-Rad), and specific primers: 5’-ACCTCTTTGACCCTGAGTTWGACA -3’ (sense oligonucleotide); 5’- CTGAAGGAGACAGGTGGAGATGA -3’ (antisense oligonucleotide); 5’- Hex-TGCCTTGACGATCTT-MGB -3’ (probe) as describe by (Zhuang et al., 2018). The thermal cycling reaction conditions were as follows: reverse transcription at 50 °C for 20 minutes, initial denaturation at 95 °C for 5 minutes, followed by 45 amplification cycles of 95 °C for 15 seconds and 60 °C for 45 seconds. Viral RNA levels were expressed on a Log_10_ scale as FFU equivalents per microgram of RNA. To estimate the viral burden, the data were normalized to a standard curve generated from serial 10-fold dilutions of viral RNA derived from known quantities of infectious SFTSV. All q-RT-PCR assays were performed in technical triplicate for each sample, with each plate containing its own standard curve. Therefore, each plate had a distinct quantification cycle (Cq) threshold, defined by the highest Cq value in the corresponding standard curve. A sample was considered positive for viral RNA if at least two of the technical replicates exhibited clear amplification curves, and the mean Cq value was below the Cq threshold derived from the standard curve for that specific q-RT-PCR plate.

### 2.6 Detection of infectious virus in nymph bodies

A subset of nymph body samples from Experiment II that exhibited the highest viral RNA levels by q-RT-PCR underwent a virus propagation assay to evaluate whether infectious virus was detectable in these samples. Nymph body samples from the mock-infected tick group were run in parallel to verify the absence of infectious virus. Vero E6 cells were seeded in 12-well plates at 1.3 x 10^5^ cells/well and incubated overnight to reach 70–85% confluency the next day, at which point the plates were then transferred into the BSL-3 facility. The culture medium (DMEM supplemented with 10% FBS and 1% penicillin-streptomycin) was gently removed, and 250 µL of clarified tick homogenate was added to duplicate wells of the plate for each sample. Plates were incubated at 37 °C with 5% CO_2_ for 1 hour with gentle rocking every 15 minutes to promote uniform viral adsorption and prevent cell monolayer desiccation. After the 1-hour incubation, inoculum was removed and each well was gently washed twice with 1 mL of PBS. Subsequently, 1 mL of fresh DMEM (supplemented with 2% FBS, 1% penicillin-streptomycin, and 2.5 ng/mL amphotericin B) was added to each well. The plates were then incubated at 37 °C with 5% CO_2_, and cells were monitored daily for the appearance of cytopathic effects (CPE). As a positive control, SFTSV stock with defined titer was added to separate wells at two different dilutions. For the negative control, wells received culture medium. All samples were analyzed in technical duplicates. Supernatant was harvested when a sample’s CPE reached at least 70%, or at 12 d.p.i., whichever came first.

### 2.7 16S rRNA amplicon sequencing of *Haemaphysalis longicornis* nymphs

Unfed, uninfected *H. longicornis* nymphs reared from larvae that had fed on either guinea pigs or mice were subjected to 16S rRNA gene sequencing. A total of five samples per host species were processed and sequenced, with each sample consisting of 15 pooled nymphs. Genomic DNA was extracted according to the manufacturer’s instructions using the DNeasy Blood & Tissue Kit (Qiagen). DNA concentration was measured using the Qubit 4.0 Fluorometer (Thermo Fisher Scientific) to ensure adequate yield and purity. Samples containing a total of 700 ng of genomic DNA in 35 µL (20 ng/µL) were submitted to GENEWIZ (Azenta Life Sciences) for 16S rRNA gene sequencing.

#### 2.7.1 Library preparation and sequencing

Next-generation sequencing library preparations and Illumina MiSeq sequencing were conducted by GENEWIZ (Azenta Life Sciences). For amplicon generation, 30–50 ng of DNA was used with a MetaVx Library Preparation kit. The V3, V4, and V5 hypervariable regions of prokaryotic 16S rRNA were targeted for taxonomic analysis, using proprietary primers designed by GENEWIZ. The relevant sequences for amplification included specific forward and reverse primers for each region. Following the first round of PCR, indexed adapters were added to the 16S rRNA amplicons to create indexed libraries suitable for NGS. These libraries were validated with an Agilent 2100 Bioanalyzer and quantified using the Qubit 2.0 Fluorometer. The multiplexed libraries were then loaded onto the Illumina MiSeq instrument, and sequencing was performed in a 2 x 300/250 paired-end configuration, with image analysis and base calling managed by the MiSeq Control Software.

#### 2.7.2 Bioinformatics analysis

Raw FASTQ reads were analyzed using QIIME2 (Version 2024.10) Amplicon platform. Residual Barcodes and primer sequences were cleaned using cut adapt tools in the QIIME2 platform. DADA2 plugin was used to denoise, filter chimeric reads, merge paired-end reads, and extract accurate amplicon sequencing variants (ASVs). The representative ASV sequences were classified using a pre-trained Naive Bayes Classifier against SILVA 138.1 reference database targeting V3–V5 hypervariable region. The ASV feature table was then used for further taxonomic analysis at the genus level of the microbial community. The feature table and taxonomic table generated in QIIME2 was used in R (Version 4.3) with modified script for further downstream analysis. Archaea, mitochondria, and chloroplasts were removed from the sequence analysis (Gil et al., 2020; Thanchomnang et al., 2023; Martinez-Villegas et al., 2024). Samples that appeared as unclassified in the SILVA database were named as “unassigned”. The relative abundance plot was generated to visualize top genera present in the samples, and these were later filtered to remove the highly abundant genus (*Coxiella*) from the plot to clearly visualize rest of the genera. The rare taxa that grouped together appeared in the abundance plots were designated as “others”. Sample complexity was analyzed using alpha and beta diversity analyses. Alpha diversity metrics measured Chao1 and Shannon diversity among samples in each experimental group to observe richness and evenness within each sample. Beta-diversity measure was assessed using Bray-Curtis distance matrix to plot PCoA (Principal coordinate Analysis) to assess differences in microbial composition between the samples.

### 2.8 Statistical analyses

To compare differences in viral RNA levels in tick or mouse samples between Experiments I–III or between tick tissue types, data were first assessed for normality using GraphPad Prism (version 9). All datasets were non-normally distributed. Thus, a Kruskal-Wallis test followed by Dunn’s multiple comparison test was used. Statistical significance was considered as *P* < 0.05. To compare overall rates of viral RNA detection in tick bodies and in tick legs between Experiments I–III, Chi-Square tests of independence were performed using GraphPad Prism; however, due to low expected frequencies in some cells, the assumptions of Chi-Square were violated and the Fisher’s Exact Test was instead utilized. The null hypothesis stated that no significant (ns) difference exists in viral RNA detection rates in tick bodies/legs between experiments.

## 3 Results

### 3.1 Acquisition of SFTSV by *Haemaphysalis longicornis* nymphs fed upon viremic mice

Three independent *in vivo* tick feeding experiments were conducted over the course of one year, with slight variations in time points of tick collection and sample processing protocols (**Figure 1**). In all experiments, *H. longicornis* nymphs were fed upon BALB/c mice that were either infected with SFTSV or were mock-infected. Experiments I, II, and III (**Figure 1**) were conducted with the same objective and rationale and were repeated three times to ensure the rigor and reproducibility of the findings. The overarching objective of the experiments was to define the temporal dynamics of SFTSV in *H. longicornis* nymphs by evaluating tick acquisition of virus via blood feeding and subsequent dissemination of virus beyond the tick midgut.

**Figure 2:**
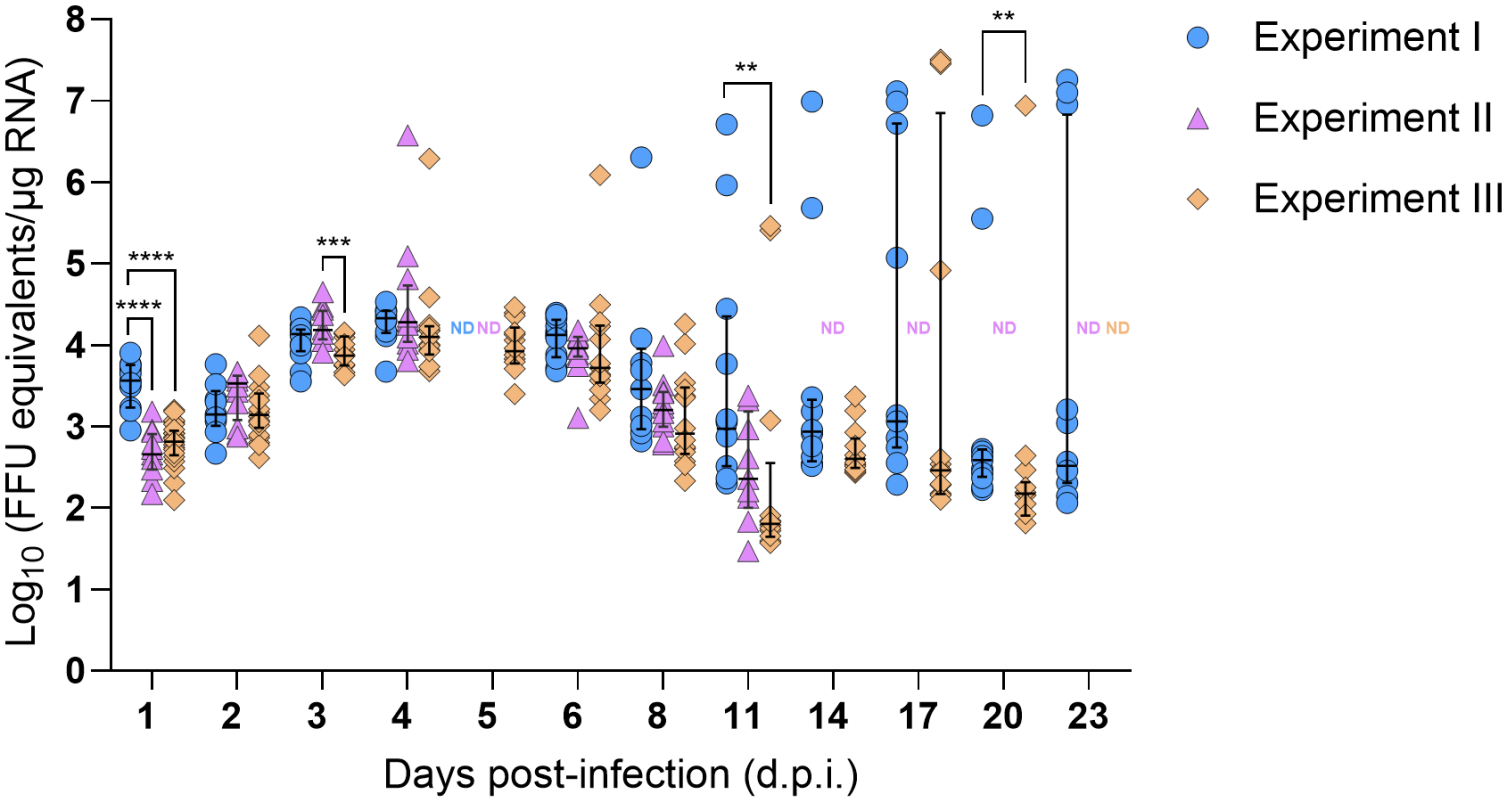
SFTSV RNA levels in bodies of *Haemaphysalis longicornis* nymphs fed on viremic mice. Viral RNA was quantified by q-RT-PCR in tick bodies collected at multiple time points during and after ticks fed on SFTSV-infected mice. Data from three independent experiments are presented. Each symbol represents an individual tick and only positive samples are plotted. Bars indicate the median and interquartile range. Viral RNA levels are expressed as FFU equivalents/µg RNA after normalization to a standard curve generated from serial 10-fold dilutions of viral RNA derived from known quantities of infectious SFTSV. Statistical analyses were performed for each time point using ANOVA (Kruskal–Wallis test) followed by Dunn’s multiple comparison test. ND: no data (i.e., no ticks collected at this time point). ****P ≤ 0.0001; ***P ≤ 0.001; **P ≤ 0.01.

SFTSV RNA levels in mouse blood were evaluated daily from 1–5 d.p.i. for mice in all three experiments (**Supplementary Figure S1**). Viral RNA levels peaked slightly at 3 d.p.i. and then gradually decreased; however, even at 5 d.p.i. (at the time of mouse euthanasia), median viral RNA levels were ≥ 4.14 Log_10_(FFU equivalents/µg RNA). At 1, 2, and 4 dpi, there were no statistically significant differences in blood viral RNA levels among mice in the three experiments; however, at 3 d.p.i., the median viral RNA level of mouse blood samples from Experiment II was significantly lower than the blood samples from Experiment III (*P*-value = 0.04), and at 5 d.p.i., the median viral RNA level of mouse blood samples from Experiment I was significantly lower than the blood samples from Experiment II (*P*-value = 0.03) (**Supplementary Figure S1**). No viral RNA was detected in the blood of mock-infected mice at any time point. Nymph bodies (with legs severed) collected at various time points across Experiments I–III were used to assess tick acquisition of virus via blood feeding (**Figure 2**). As expected, no viral RNA was detected in any of the nymph bodies fed upon mock-infected mice. However, for *H. longicornis* nymphs fed upon SFTSV-infected mice, viral RNA was detected in bodies of nymphs as early as 1 d.p.i., indicating that virus acquisition can occur within the first 24 hours of feeding on viremic mice. Across all three experiments, viral RNA was consistently detected in the bodies of *H. longicornis* nymphs at every time point during partial feeding on SFTSV-infected mice (1–2 d.p.i.), as well as throughout the pre-molt period (3–23 d.p.i.) (**Figure 2**). The rate of detection of SFTSV RNA in tick bodies remained high at each time point across the three experiments (**Table 1**). Additionally, the Fisher’s Exact Test revealed no statistically significant differences in overall viral RNA detection rates (across all time points) in nymph bodies among any of the experiments.

**Table 1:** Rate of detection of SFTSV RNA by q-RT-PCR in bodies of *Haemaphysalis longicornis* nymphs.

Days post-infection (d.p.i.)	Rate of SFTSV RNA detection in *H. longicornis* nymph bodies by q-RT-PCR (# positive/total # screened)		
	Experiment I	Experiment II	Experiment III
1	100% (11/11)	100% (12/12)	100% (25/25)
2	100% (9/9)	100% (12/12)	100% (22/22)
3	100% (12/12)	100% (12/12)	100% (18/18)
4	100% (10/10)	100% (12/12)	100% (18/18)
5	ND	ND	100% (14/14)
6	100% (12/12)	100% (12/12)	100% (14/14)
8	100% (9/9)	100% (12/12)	100% (14/14)
11	100% (12/12)	75% (9/12)	100% (14/14)
14	100% (12/12)	ND	92.9% (13/14)
17	100% (11/11)	ND	100% (14/14)
20	100% (12/12)	ND	100% (14/14)
23	100% (12/12)	ND	ND
Total across all timepoints	100% (122/122)	96.4% (81/84)	99.4% (180/181)

ND, no data (i.e., no ticks collected at this time point).

Viral RNA levels were also quantified and compared in nymph bodies collected at each time point across the three experiments. Interestingly, SFTSV RNA levels increased progressively over time, with medians peaking at 4 d.p.i. (**Figure 2**). Some individual nymphs exhibited markedly elevated SFTSV RNA levels, with initial detection of such highly positive individual nymphs occurring as early as 4 d.p.i. in Experiments II and III. These individual ticks with high viral RNA levels were identified during the pre-molt phase, particularly between 8–23 d.p.i. in Experiment I, and at 4, 6, 11, 17, and 20 d.p.i. in Experiment III. Generally, at each time point, there were not statistically significant differences in the median viral RNA levels in nymph bodies compared across the three experiments, with the exception of the 1, 3, 11 and 20 d.p.i. time points.

To verify that detection of viral RNA in nymph bodies also corresponds to the detection of infectious virus, a virus propagation assay was applied to a subset of nymph bodies generated in Experiment II. Nymph samples collected from Experiment II were not transferred directly into TRIzol but were homogenized in media first, which enabled the virus propagation assay to be applied to the nymph bodies. A subset of 5–7 nymph bodies that exhibited the highest SFTSV RNA levels (**Figure 2**) at experimental time points ranging from 1–11 d.p.i. were selected for the virus propagation assay. Additional nymph bodies from the mock-infected mice were included in the virus propagation assay as controls. Nymph bodies harvested at 4 d.p.i. had the highest viral RNA levels, and CPE was detected in all seven (100%) of these samples (**Table 2**). Additionally, five of the six (83.3%) nymph body samples harvested at 3 d.p.i. resulted in detectable CPE using the virus propagation assay. No CPE was detected in any nymph bodies derived from nymphs that fed upon mock-infected mice, or in nymph bodies fed upon SFTSV-infected mice that were harvested at 1 and 11 d.p.i. (**Table 2**).

**Table 2:** Propagation of SFTSV from *Haemaphysalis longicornis* nymph bodies that screened positive for viral RNA by q-RT-PCR.

d.p.i. at which nymph bodies were harvested from *in vivo* Experiment II	Average Log_10_ (FFU equivalents/µg RNA) by q-RT-PCR for nymph bodies	Rate of CPE detection in nymph bodies (# positive/total # screened)	Days required for nymph bodies to reach ≥ 70% CPE
1	2.95	0% (0/5)	NA
2	3.63	20% (1/5)	8
3	4.42	83.3% (5/6)	7–9
4	5.76	100% (7/7)	6–12
6	4.10	20% (1/5)	8
8	3.61	20% (1/5)	8
11	3.08	0% (0/5)	NA

CPE, cytopathic effect.

NA, not applicable (no CPE detected).

### 3.2 Dissemination of SFTSV beyond the midgut of *Haemaphysalis longicornis* nymphs

**Figure 3:**
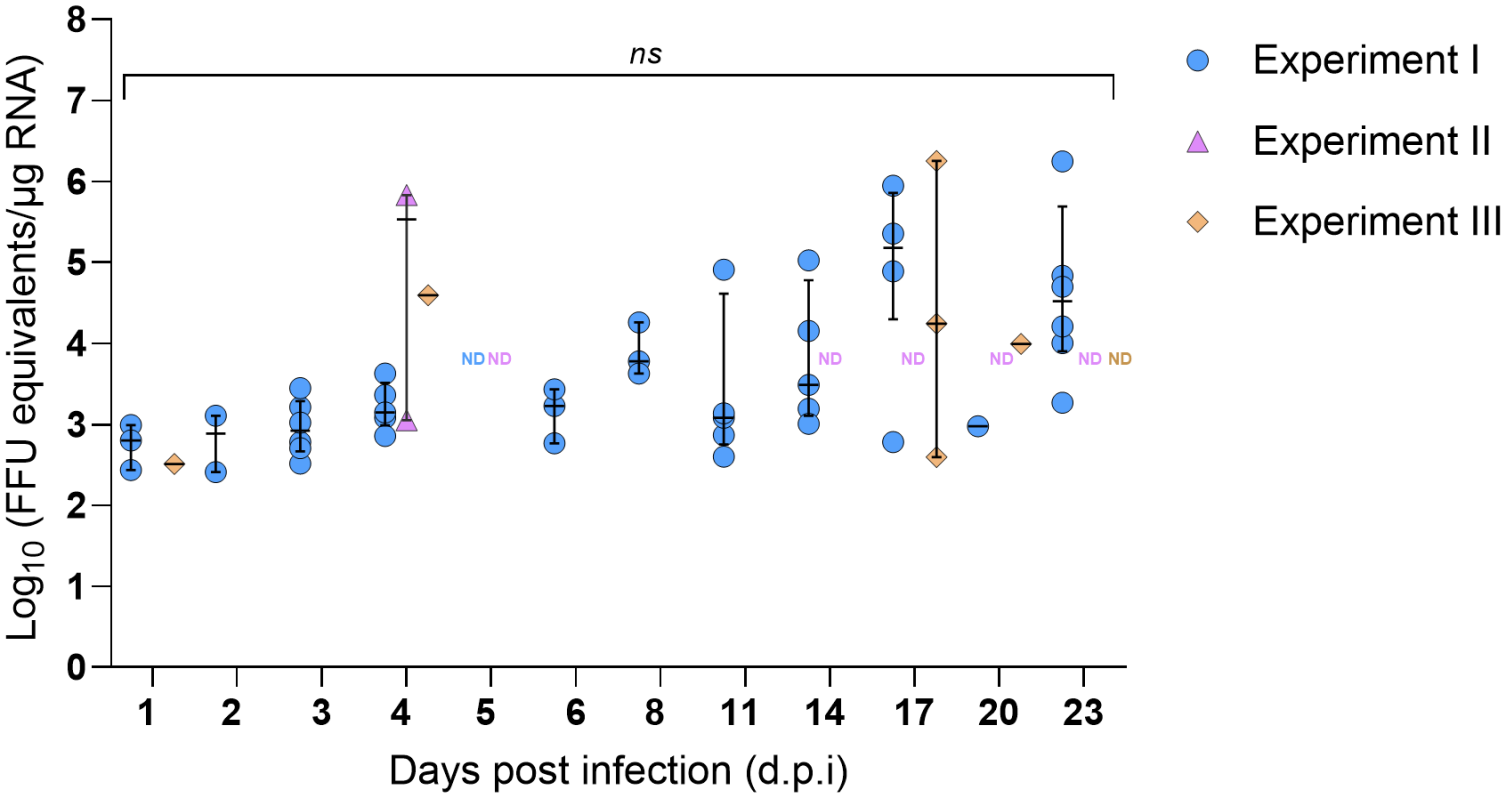
SFTSV RNA levels in legs of *Haemaphysalis longicornis* nymphs fed on viremic mice. Viral RNA was quantified by q-RT-PCR in tick legs collected at multiple time points during and after ticks fed on SFTSV-infected mice. Data from three independent experiments are presented. Each symbol represents an individual tick and only positive samples are plotted. Bars indicate the median and interquartile range. Viral RNA levels are expressed as FFU equivalents/µg RNA after normalization to a standard curve generated from serial 10-fold dilutions of viral RNA derived from known quantities of infectious SFTSV. Statistical analyses were performed for each time point using ANOVA (Kruskal–Wallis test) followed by Dunn’s multiple comparison test. ND: no data (i.e., no ticks collected at this time point). *ns*: indicates no statistical significance.

Due to the open circulatory system of arthropods, nymph legs were used as a proxy for hemolymph to assess the kinetics of SFTSV dissemination beyond the tick midgut. The first detection of SFTSV RNA in the legs of *H. longicornis* nymphs occurred at 1 d.p.i. (Experiments I and III), indicating that virus had already disseminated beyond the midgut and suggesting early systemic spread within the tick (**Figure 3**). SFTSV-positive nymph legs were detected across all time points (1–23 d.p.i.) in Experiment I, with viral RNA detection rates ranging between 8.3–50% (**Table 3**). In contrast, few nymph leg samples from Experiments II and III screened positive for viral RNA (**Table 3**; **Figure 3**). SFTSV-positive legs were only detected at 4 d.p.i. in Experiment II and at 1, 4, 17 and 20 d.p.i. in Experiment III (**Figure 3**). To compare overall rates of viral RNA detection (across all time points) in nymph legs among Experiments I–III, the Fisher’s Exact Test was performed, revealing a statistically significant difference in rates of viral RNA detection in nymph legs from Experiment I versus Experiment II (*P*-value < 0.0001) and in legs from Experiment I versus Experiment III (*P*-value < 0.0001). However, there was no statistically significant difference in the rates of viral RNA detection in nymph legs from Experiments II and III (*P*-value > 0.9999). As expected, viral RNA was not detected in nymph legs that had fed on mock-infected mice in any of the three experiments.

**Table 3:** Rate of detection of SFTSV RNA by q-RT-PCR in legs of *Haemaphysalis longicornis* nymphs.

Days post-infection (d.p.i.)	Rate of SFTSV RNA detection in *H. longicornis* nymph legs by q-RT-PCR (# positive/total # screened)		
	Experiment I	Experiment II	Experiment III
1	27.3% (3/11)	0% (0/12)	4% (1/25)
2	22.2% (2/9)	0% (0/12)	0% (0/22)
3	50% (6/12)	0% (0/12)	0% (0/18)
4	50% (5/10)	16.7% (2/12)	5.6% (1/18)
5	ND	ND	0% (0/14)
6	25% (3/12)	0% (0/12)	0% (0/14)
8	33.3% (3/9)	0% (0/12)	0% (0/14)
11	41.7% (5/12)	0% (0/12)	0% (0/14)
14	41.7% (5/12)	ND	0% (0/14)
17	36.4% (4/11)	ND	21.4% (3/14)
20	8.3% (1/12)	ND	7.1% (1/14)
23	50% (6/12)	ND	ND
Total across all timepoints	35.2% (43/122)	2.4% (2/84)	3.3% (6/181)

ND, no data (i.e., no ticks collected at this time point).

**Figure 4:**
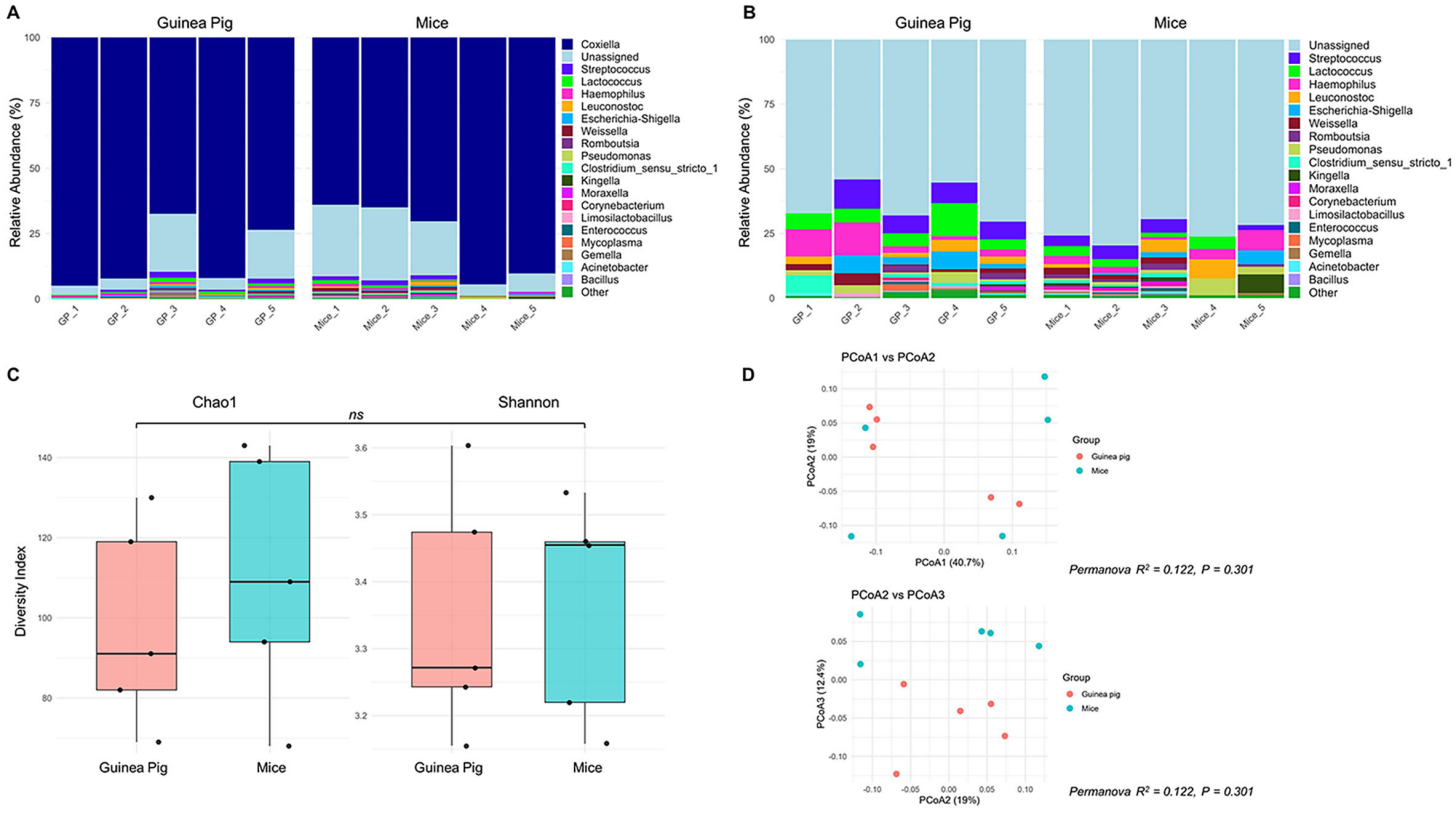
Bacterial community composition analysis by 16S rRNA sequencing of *Haemaphysalis longicornis* nymphs reared from larvae fed on guinea pigs or mice **(A)** Relative abundance plot of top 20 genera per sample, where the genus *Coxiella* is highly abundant; **(B)** Filtered relative abundance plot excluding *Coxiella*; **(C)** Alpha diversity plot including Chao1 and Shannon indices to observe the richness and evenness of the bacterial community of nymphs that fed as a larvae on guinea pigs or mice; **(D)** Beta-diversity plot of Principal coordinate analysis (PCoA1 vs PCoA2, and PCoA2 vs PCoA3) based on Bray-Curtis metrics to measure the abundance-based bacterial differences among ticks fed on guinea pigs or mice. *ns*: indicates no statistical significance; R2 (Coefficient of determination) value close to zero = not significant.

The lower overall rates of viral RNA detection in tick legs from Experiments II and III compared to Experiment I (**Table 3**) prompted careful consideration of any potential experimental design differences among these three experiments that could explain the unique patterns of SFTSV dissemination beyond the tick midgut. A difference identified in experimental design was the choice of pathogen-free vertebrate host (guinea pigs or mice) used to feed larvae in the *H. longicornis* colony in order to generate nymphal ticks that were used in Experiments I–III. Specifically, the nymphal *H. longicornis* used in Experiments I and II were fed as larvae on guinea pigs, whereas the nymphs used in Experiment III were fed as larvae on mice. Thus, 16S rDNA gene sequencing was performed on unfed, pathogen-free *H. longicornis* nymphs from two groups, one reared from larvae fed on guinea pigs and the other reared from larvae fed on mice, to evaluate differences in bacterial microbiota in the unfed nymphs. The *Coxiella* genus was most abundant across both groups of nymphs (**Figure 4A**) (Wang et al., 2018); therefore, *in silico* removal of *Coxiella* sequences was performed (**Figure 4B**). There were no statistically significant differences in the bacterial species richness or evenness within samples (**Figure 4C**), or differences in bacterial composition among samples (**Figure 4D**), derived from the two groups of nymphs fed as larvae on different vertebrate hosts.

Another experimental design difference observed was that the post-molting age of nymphs differed among the experiments. The post-molting age of nymphs used in Experiments I and III was 2.5 months, whereas the post-molting age of nymphs used in Experiment II was 4 months. Although Experiments I and II used nymphs of different ages post-molting and statistically significant differences in viral RNA detection rates were observed in nymph legs from Experiment I versus Experiment II (**Table 3**), there were also statistically significant differences in viral RNA detection rates in nymph legs from Experiment I versus Experiment III (**Table 3**), yet both of these experiments (I and III) used nymphs of the same age.

Despite the lower overall rate of viral RNA detection in tick legs from Experiments II and III, individual ticks with SFTSV RNA levels greater than 4 Log_10_(FFU equivalents/µg RNA) in their legs were observed as early as 4 d.p.i. in both experiments, and also at 17 d.p.i. in Experiment III (**Figure 3**). In Experiment I, the majority of leg samples that screened positive at 17 and 23 d.p.i. had viral RNA levels greater than 4 Log_10_(FFU equivalents/µg RNA). Additionally, in Experiment II, a nymph with 5.83 Log_10_(FFU equivalents/µg RNA) of viral RNA detected in its legs at 4 d.p.i. was further analyzed by virus propagation and focus-forming assay (FFA). Infectious SFTSV was detected and tittered in the body (4.74 Log_10_[FFU/mL]) and legs (3.45 Log_10_ [FFU/mL]) of this individual tick, providing evidence of infectious SFTSV disseminating beyond the midgut of this tick. Nymphs derived from mock-infected mice were also screened for infectious SFTSV, and all of the body and leg samples from these mock-infected ticks screened negative for virus.

### 3.3 Correlation of viral RNA levels in *Haemaphysalis longicornis* nymph bodies and legs

Using representative data from Experiment I, the correlation between viral RNA levels in each tick’s body (as a measure of virus acquisition) and legs (as a measure of dissemination) was assessed (**Supplementary Figure S2A**). The analysis revealed a moderate statistically significant positive correlation (Spearman’s r = 0.4217, *P* < 0.0001), indicating that higher levels of viral RNA in nymph bodies were associated with increased viral RNA levels in the nymph legs (**Supplementary Figure S2B**). Although this correlation was positive (r = 0.4217), it was nonlinear. This finding supports the notion that as viral replication intensifies at the site of acquisition, dissemination to peripheral regions such as the legs occurs concurrently, reflecting systemic spread within the tick.

### 3.4 Transstadial transmission of SFTSV within *Haemaphysalis longicornis*

**Figure 5:**
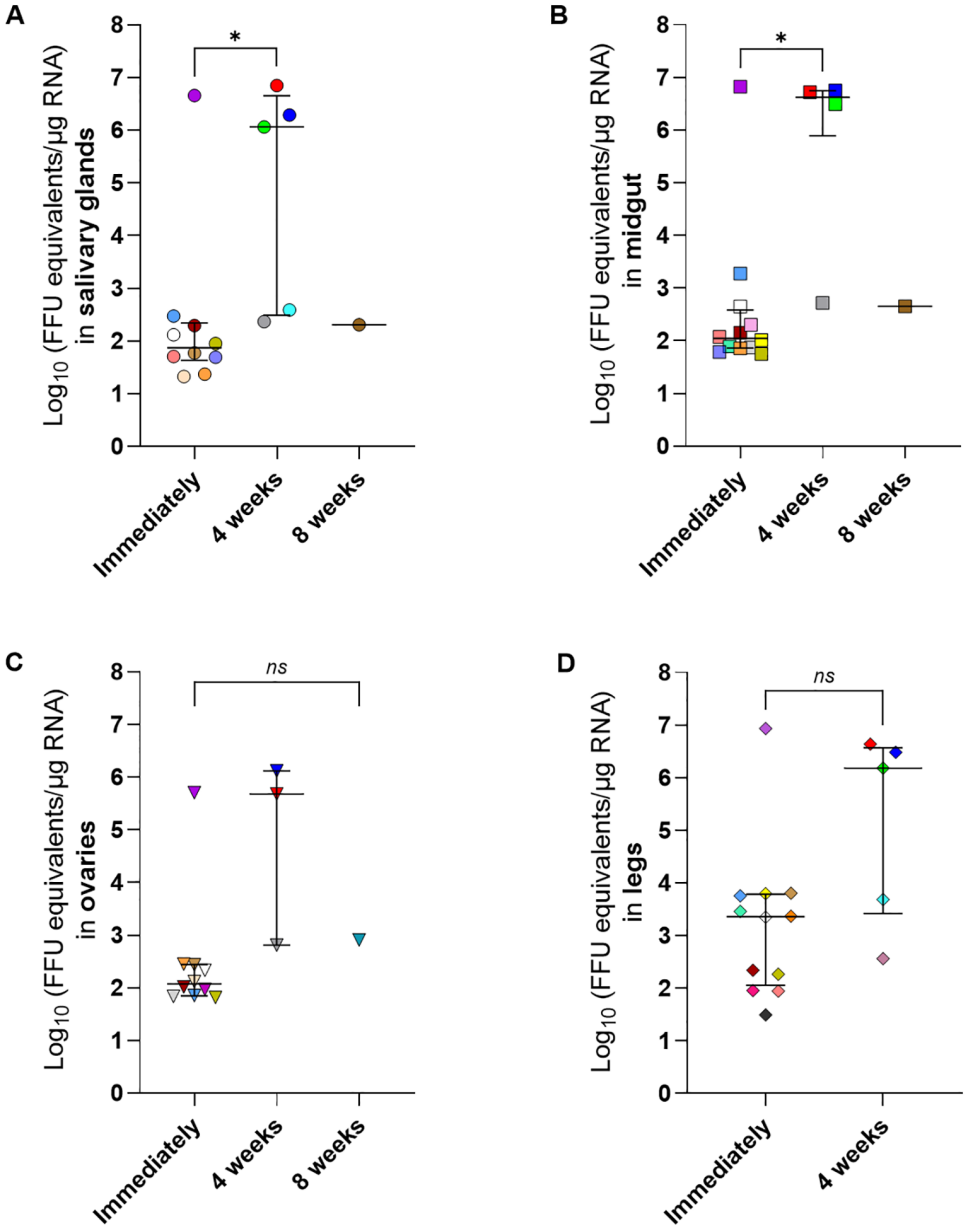
SFTSV RNA levels in dissected tissues of *Haemaphysalis longicornis* molted adults that fed on viremic mice as nymphs. Adult ticks that had acquired SFTSV while feeding on viremic mice during the nymphal life cycle stage were dissected after molting. Tissues were collected at three time points: immediately after molting (Immediately); 4 weeks or 8 weeks post-molt. Viral RNA levels were measured using q-RT-PCR in the following tissues: **(A)** salivary glands, **(B)** midgut, **(C)** ovaries and **(D)** legs. Each symbol color represents an individual tick, and the same color is used to denote the other tissues derived from that tick across all panels. Only positive samples are plotted. Bars indicate the median and interquartile range. Viral RNA levels are expressed as FFU equivalents/µg RNA after normalization to a standard curve generated from serial 10-fold dilutions of viral RNA derived from known quantities of infectious SFTSV. Only positive samples are shown on the graph. Statistical analysis was performed using ANOVA (Kruskal–Wallis test) followed by Dunn’s multiple comparison test. *ns*: indicates no statistical significance. *P< 0.05.

To assess the transstadial transmission of SFTSV, fully engorged *H. longicornis* nymphs that had naturally fed upon and detached from viremic or mock-infected mice were maintained in an ACL3 facility under controlled temperature and humidity until molting into adults (**Figure 1**). Salivary glands, midgut, ovaries, and legs were harvested from each adult tick at three time points post-molt: immediately after molting, four weeks post-molt, and eight weeks post-molt. SFTSV RNA was detected at all post-molt time points analyzed, providing evidence of transstadial transmission of the virus from the nymphal to the adult tick life cycle stage (**Figures 5A–D**). There were no statistically significant differences in the median viral RNA levels when the four different types of tick tissues were compared within each time point; however, when the median viral RNA levels were compared for each type of tick tissue across time points, there were significantly higher levels of viral RNA detected in the tick salivary glands (**Figure 5A**) (*P*-value = 0.0273) and midguts (**Figure 5B**) (*P*-value = 0.0419) at 4 weeks post-molt compared to immediately post-molt. No statistical differences were observed in the median viral RNA levels of ovaries and legs across time points (**Figures 5C, D**) The rate of detection of viral RNA in tissues of *H. longicornis* adults was highest when ticks were dissected immediately after molting (**Table 4**). However, these rates of viral RNA detection decreased over time for each type of tissue. By eight weeks post-molt, the low rate of detection of viral RNA in tick tissues was most pronounced, with only 6.67% positivity in the salivary glands, midgut, and ovaries, and absence of SFTSV RNA in legs. As expected, no viral RNA was detected in adult tissues from nymphs that had fed on mock-infected mice.

**Table 4:** Rate of detection of SFTSV RNA by q-RT-PCR in dissected tissues of *Haemaphysalis longicornis* adults after molting.

Post-molt tick dissection and tissue harvest time points	Rate of SFTSV RNA detection in dissected tissues of *H. longicornis* by q-RT-PCR (# positive/total # screened)			
	Salivary glands	Midgut	Ovaries	Legs
Immediately	62.5% (10/16)	75% (12/16)	62.5% (10/16)	75% (12/16)
4 weeks	55.6% (5/9)	44.4% (4/9)	50% (3/6)	55.6% (5/9)
8 weeks	6.7% (1/15)	6.7% (1/15)	6.7% (1/15)	0%

## 4 Discussion

In ticks that are competent vector species, viruses acquired during blood feeding must overcome a series of anatomical and immunological barriers before they can establish infection, disseminate within the tick, and ultimately be transmitted to a new vertebrate host during a subsequent blood meal taken by the tick (Nuttall et al., 1994; Nuttall, 2013). Upon ingestion during blood feeding, the first tick interface encountered by a virus is the peritrophic membrane, a chitinous, semi-permeable matrix that surrounds the ingested bloodmeal and restricts pathogen access to the midgut epithelial cells (Sonenshine and Hynes, 2008). Following entry into the tick midgut, viruses are exposed to a complex array of innate immune effectors, including antimicrobial peptides, digestive proteases, protease inhibitors, opsonins, reactive oxygen species, and other cytotoxic molecules, that collectively act to restrict pathogen survival and colonization of the midgut epithelial cells (Sonenshine and Hynes, 2008; Fogaca et al., 2021). Successful transmission by competent tick species requires that viruses cross the midgut barrier to access the hemolymph in a step that is critical for systemic dissemination within the tick. Hemolymph, which circulates throughout the tick’s body cavity and reaches all internal tissues and organs, facilitates the spread of viruses to distal sites such as the salivary glands. However, hemolymph also has a functional immune system, composed of humoral and cellular components, which act to detect and eliminate invading pathogens (Hajdusek et al., 2013). From the hemolymph, viruses must invade the salivary glands in order to be transmissible to a vertebrate host during blood feeding at a subsequent life cycle stage. An additional requirement for horizontal transmission of virus to occur is that the virus must maintain infectivity across life cycle stages of the tick. This requires the virus to persist through molting events, which are marked by histolytic enzyme activity and extensive tissue remodeling, creating an adverse environment for virus survival (Kaufman, 2013; Nuttall, 2013). With all of these anatomical barriers and immunological intricacies, it is critical that specific virus-tick species pairings are carefully examined in order to elucidate the intra-tick infection dynamics that enable specific viruses to infect, disseminate, and persist within a particular tick species.

*Haemaphysalis longicornis* is the primary vector of SFTSV. As an invasive species now established in multiple regions beyond its native range of East and Southeast Asia, this tick has garnered increasing attention due to its role in the transmission of SFTSV. In order to develop rational intervention strategies that disrupt SFTSV maintenance and transmission by *H. longicornis* in nature, and thereby reduce the risk of human exposure, it is essential to first elucidate the fundamental virus infection dynamics within the tick vector. The main goal of this study was to define the intra-tick kinetics of SFTSV acquisition and virus dissemination beyond the tick midgut.

The dynamics of SFTSV acquisition by *H. longicornis* nymphs fed on viremic mice were temporally examined by screening partially-fed and fully-engorged pre-molt nymphs for SFTSV (**Figure 1**). Across three independent experiments, a consistent pattern of SFTSV detection was observed in partially-fed and fed pre-molt nymphs (**Table 1**, **Figure 2**). In Experiments I–III, viral RNA was detected in the bodies of partially-fed *H. longicornis* nymphs as early as 1 d.p.i. (the first time point assessed), indicating rapid tick acquisition of SFTSV during blood feeding on viremic mice. Additionally, the rate of detection of SFTSV RNA in nymph bodies remained consistently high across all experiment time points (**Table 1**), indicating a sustained viral presence during both the partially-fed and fed pre-molt phases. At each time point, viral RNA levels in nymph bodies remained relatively consistent across Experiments I–III. Notably, some variation in SFTSV RNA levels among individual ticks within the same time points was observed, particularly from 11 to 23 d.p.i. in Experiment I and at 17 and 20 d.p.i. in Experiment II. Such variation can be observed in *in vivo* experiments and likely reflects natural biological heterogeneity among individual ticks. Statistically significant differences in median viral RNA levels in nymph bodies among experiments were observed at only four time points: 1, 3, 11, and 20 d.p.i. (**Figure 2**). The viral RNA levels in mouse blood were assessed at time points ranging from 1–5 d.p.i. to determine whether differences in mouse viremia among the three experiments could account for the significantly different levels of viral RNA detected in nymph bodies at 1 and 3 d.p.i. Ultimately, despite significantly lower SFTSV RNA levels in mouse blood at 3 d.p.i. in Experiment II compared to Experiment III (**Supplementary Figure S1**), viral acquisition by nymphs in Experiment II at the 3 d.p.i. time point was not negatively affected (**Figure 2**). Furthermore, temporal assessment of viral RNA levels in the partially-fed and fed pre-molt nymphal ticks demonstrated that the median viral RNA levels in nymph bodies increased progressively after initial detection at 1 d.p.i., peaking at 4 d.p.i. (**Figure 2**). This steady increase in median viral RNA levels detected within the nymphs over time may reflect enhanced viral replication within the tick body. To address this, nymph bodies from Experiment II were individually homogenized then fractioned and screened for both viral RNA via q-RT-PCR (**Table 1**, **Figure 2**) and for the presence of infectious virus via virus propagation assay. The virus propagation assay confirmed the presence of infectious virus in nymph body samples as early as 2 d.p.i. and through 8 d.p.i. (**Table 2**), as measured by the detection of CPE in Vero E6 cells infected with nymph body homogenate. The rate of CPE detection in nymph body samples was highest at the 3 and 4 d.p.i. time points, with 83.3% and 100% of the samples screening positive for CPE, respectively (**Table 2**). These results, combined with the highest median viral RNA levels in nymph bodies being detected at 4 d.p.i. (**Figure 2**), suggest that viral replication was possible within fed pre-molt nymphs at this time point.

In this study, SFTSV RNA was also detected in tissues of molted *H. longicornis* adults that had acquired the virus as nymphs while feeding on viremic mice, demonstrating transstadial transmission of SFTSV (**Table 4**, **Figure 5**). The detection rate of viral RNA in adult tick tissues was higher in samples collected immediately after molting compared to those collected four or eight weeks after molting (**Table 4**). This suggests a marked reduction in viral RNA persistence in tick tissues as time progresses following the molt. However, in the ticks that were capable of maintaining SFTSV from the nymphal to adult life cycle stage, median viral RNA levels in adult tick salivary glands and midguts were significantly higher at four weeks post-molt compared to the viral RNA levels in these tissues when ticks were dissected immediately after molting (**Figure 5**). This pattern suggests that while transstadial maintenance of the virus occurs, the window for efficient horizontal transmission may be greatest four weeks after molting when viral load in key tissues such as the salivary glands is highest. These findings provide insight toward developing future intervention strategies targeting tick-to-host horizontal transmission of SFTSV.

In previous studies assessing *H. longicornis* vector competence for SFTSV and vertebrate amplifying host status, high rates of detection of SFTSV in engorged nymphs fed upon viremic mice or hedgehogs, as well as transstadial transmission of virus from the engorged nymphs to adults, were reported (Luo et al., 2015; Zhao et al., 2022). The high rates of SFTSV detected in partially-fed and fully-engorged *H. longicornis* nymphs from the present study align with previously published findings whereby 100% of naïve *H. longicornis* nymphs fed upon viremic mice or hedgehogs acquired SFTSV (Luo et al., 2015; Zhao et al., 2022). However, this study is unique in that it systematically evaluated *H. longicornis* nymphs at various time points during/after feeding on viremic mice for the presence of SFTSV (both viral RNA and infectious virus) in order to provide key insights into the temporal pattern of virus acquisition and replication. Moreover, the present study’s confirmation of transstadial maintenance of SFTSV by *H. longicornis*, coupled with the temporal assessment of SFTSV RNA levels and rates of detection, further strengthen our understanding of SFTSV persistence within the vector (Zhao et al., 2022).

Building on this study’s temporal analysis of SFTSV acquisition by *H. longicornis* nymphs and viral persistence in molted adults, the kinetics of virus dissemination beyond the nymphal midgut were examined. SFTSV dissemination into the nymph hemolymph was assessed by detecting viral RNA in nymph legs corresponding to the same nymph bodies in which viral acquisition had been detected. Due to the technical challenges of harvesting nymph hemolymph, and based on the open circulatory system of arthropods (Grubhoffer et al., 2013), legs from the nymphal ticks in the present study were used as a hemolymph proxy for assessing systemic infection within the nymphs. Detection of viral RNA in leg samples indicates that virus can escape the midgut barrier and disseminate via hemolymph to peripheral tissues, a critical step for salivary gland invasion and transmission of virus to vertebrate hosts. Although a handful of previously published studies have reported the detection of virus in tick hemolymph (Booth et al., 1991; Gonzalez et al., 1991; Dickson and Turell, 1992; Kaufman and Nuttall, 2003) these studies either involved artificially-infected ticks or failed to perform a time-based analysis of virus detection in hemolymph. The present study is unique in that it is the first to report the intra-tick kinetics of virus dissemination from midgut to hemolymph in ticks naturally infected by feeding on viremic animals. Interestingly, viral RNA was detected in several nymph legs as early as 1 d.p.i. in Experiments I and III (**Figure 3**, **Table 3**), suggesting early viral dissemination beyond the midgut can occur while nymphal ticks are still attached and feeding on the viremic host. Additional time points ranging from 2–23 d.p.i. also yielded ticks with viral RNA detected in their legs (**Figure 3**, **Table 3**). An Experiment II nymph with 5.83 Log_10_(FFU equivalents/µg RNA) of SFTSV RNA detected in its legs at 4 d.p.i. was further analyzed by virus propagation assay and FFA. The presence of infectious SFTSV in the legs of this tick was confirmed by detection of CPE and a titer of 3.45 Log_10_ (FFU/mL). Ultimately these findings provide strong evidence of active viral replication and systemic dissemination within the tick at 4 d.p.i., indicating that the virus acquired during early phases of feeding remains viable and replicates within the vector. Furthermore, in Experiment I, where SFTSV RNA was detected in nymph legs across all time points, a moderate but statistically significant positive correlation between viral RNA levels in nymph bodies and legs (r = 0.4217, *P* < 0.0001) was revealed (**Supplementary Figure S2B**), indicating that an increased systemic viral burden in the nymph body is associated with viral dissemination beyond the midgut.

In the present study, the overall rate of SFTSV RNA detection in *H. longicornis* nymph legs from Experiment I was significantly higher than the rates observed in Experiments II and III (**Table 3**), suggesting that SFTSV was capable of escaping the midgut barrier and disseminating into the hemocoel in fewer ticks derived from Experiments II and III compared to Experiment I. After carefully comparing all experimental parameters used across Experiments I–III, a difference noted was the species of vertebrate host used to feed *H. longicornis* larvae that later molted into the nymphs used in the experiments. In Experiments I and II, the nymphs originated from larvae that had fed on guinea pigs, whereas in Experiment III, the nymphs fed as larvae on mice. Notably, previous studies have demonstrated that the nymphal tick microbiome composition can be influenced by larval blood meal source (Swei and Kwan, 2017; Landesman et al., 2019), and perturbations of the gut microbiota can influence pathogen acquisition in ticks (Narasimhan et al., 2014; Narasimhan and Fikrig, 2015). This raised the possibility that factors such as host blood meal source for tick larvae may perturb the microbiota composition of nymphal ticks, which could explain the differences observed in viral dissemination rates among nymphs from Experiments I–III of the present study. However, 16S rRNA sequencing analysis of pathogen-free unfed *H. longicornis* nymphs reared as larvae on either guinea pigs or mice revealed no significant differences in bacterial species richness or evenness within samples, nor any differences in bacterial community composition between groups (**Figure 4**). Therefore, the larval host blood meal sources for the *H. longicornis* nymphs used in Experiments I–III are unlikely to explain the different SFTSV dissemination efficiencies detected among the experiments. However, future studies using SFTSV-infected nymphs fed as larvae on different host species may help to further elucidate whether host-associated microbiomes influence viral dissemination dynamics.

Another difference observed across Experiments I–III was the post-molting age of the nymphs at the time of infestation on SFTSV- or mock-infected mice. Nymphs in Experiments I and III were 2.5 months post-molt, whereas those in Experiment II were 4 months post-molt. Although Experiments I and II involved nymphs of distinct post-molting ages and showed statistically significant differences in viral RNA detection rates in nymph legs, Experiment I and Experiment III also differed significantly in leg viral RNA detection rates despite using nymphs of the same age. Furthermore, no statistically significant differences in leg viral RNA detection rates were observed between Experiments II versus III, even though the nymphs differed in age. Taken together, these findings do not provide consistent evidence that post-molting nymph age influences SFTSV acquisition or dissemination efficiency. However, it is well established that the physiological status of ticks changes over time following molting, including progressive metabolic costs and depletion of energy reserves. These factors could plausibly affect the efficiency of virus acquisition and dissemination. While no direct evidence currently exists in the literature to support this link, the possibility warrants consideration in future studies.

While findings from the present study provide insight into the kinetics of SFTSV acquisition by *H. longicornis* from a viremic vertebrate host and the timeline of virus dissemination beyond the nymph midgut, there are some limitations that should be considered. First, although viral RNA was detected in tick samples from Experiments I–III, efforts to detect infectious virus were limited to the tick samples derived solely from Experiment II. Nymph samples from Experiment II were processed in a manner that screened a fraction of each tick homogenate for viral RNA while the remaining fraction was subjected to the virus propagation assay, whereas nymph samples from Experiments I and III were homogenized directly in TRIzol and thus only enabled viral RNA detection. While the virus propagation assay performed on nymph body samples from Experiment II indeed demonstrated the presence of infectious virus particles (**Table 2**), it did not provide quantitative data to reflect the infectious units of virus in each sample. As such, the replication kinetics of virus within nymph body samples was not evaluated in the present study. However, the body and corresponding legs of a single tick from 4 d.p.i. were subjected to an FFA, ultimately providing insight into the load of infectious virus present within those samples. For the 4 d.p.i. nymph body and leg sample that underwent both viral RNA detection by q-RT-PCR and infectious virus detection by FFA, the sensitivity difference between these two assays was revealed, with the FFA yielding values that were 1.8–2.4 Log_10_ lower than the q-RT-PCR assay when the same samples were screened by both assays. Thus, given that the FFA has lower sensitivity than the q-RT-PCR assay, and that many of the nymph body and leg samples had moderate viral RNA levels (i.e., levels ranging from 2–5 Log_10_ [FFU equivalents/µg RNA]), the FFA was not applied to every tick body and leg sample derived from Experiment II. Instead, the virus propagation assay was relied upon to demonstrate infectivity of these samples. Given the sensitivity limitation of the FFA, future studies could apply endpoint dilution assays, such as the TCID_50_, as an alternative to quantify infectious virus in tick samples. Another limitation of the present study was that virus localization in specific nymph organs was not assessed. While detection of SFTSV in nymph legs, used here as a proxy for hemolymph, indicates that virus had disseminated beyond the midgut, this study did not involve dissecting and examining specific organs from partially-fed nymphs or from engorged pre-molt nymphs for the presence of virus. Therefore, the kinetics of SFTSV dissemination to organs such as the salivary glands remain unknown. Future studies will assess the dissemination pattern of SFTSV to other organs beyond the midgut and will utilize a variety of molecular and histological assays to achieve this.

In conclusion, findings from this study clearly demonstrate that SFTSV is rapidly acquired within the first 24 hours of *H. longicornis* nymphs feeding on viremic mice, and that the virus is also capable of early dissemination beyond the nymph midgut at time points during blood feeding, as well as at time points after the nymphal blood meal is complete. Transstadial transmission of SFTSV from nymphs to adults was also detected and provided evidence that time post-molt can influence the efficiency and level of virus maintained in the molted ticks. Ultimately, this was the first study to define the intra-tick kinetics of viral acquisition, as well as dissemination beyond the tick midgut for any tick-borne virus. These findings lay the foundation for defining key SFTSV-*H. longicornis* interactions that could be targeted in novel intervention strategies that disrupt virus transmission by or persistence within the tick, and thus reduce the risk of human and animal exposure.

## Data Availability

The datasets presented in this study can be found in the online repository. The names of the repository and accession number(s) can be found below: Accession to SRA data: (https://www.ncbi.nlm.nih.gov/sra/PRJNA1334343) Bioproject: PRJNA1334343 Acession number: SRR35840998, SRR35840997, SRR35840996, SRR35840995, SRR35840994, SRR35840993, SRR35840992, SRR35840991, SRR35840990, SRR35840989.
