# Supplementary figures and images for: Defining the kinetics of severe fever with thrombocytopenia syndrome virus acquisition and dissemination in naturally-infected Haemaphysalis longicornis
Source: Front Cell Infect Microbiol. 2025 Nov 3;15:1706970. doi: 10.3389/fcimb.2025.1706970 (PMC12620447; doi:10.3389/fcimb.2025.1706970)

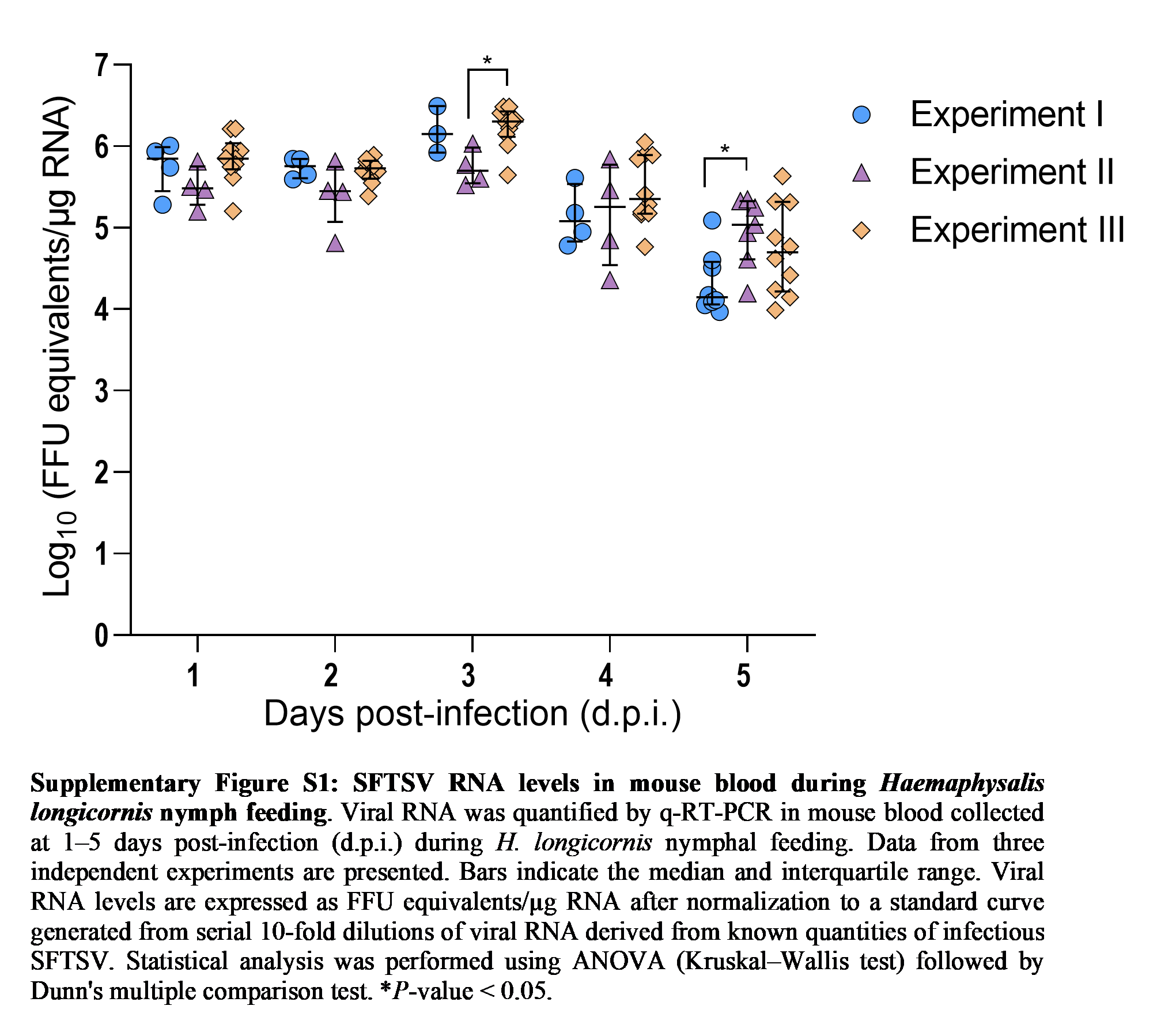

Supplement: Supplementary file 1 [file Image1.tif]

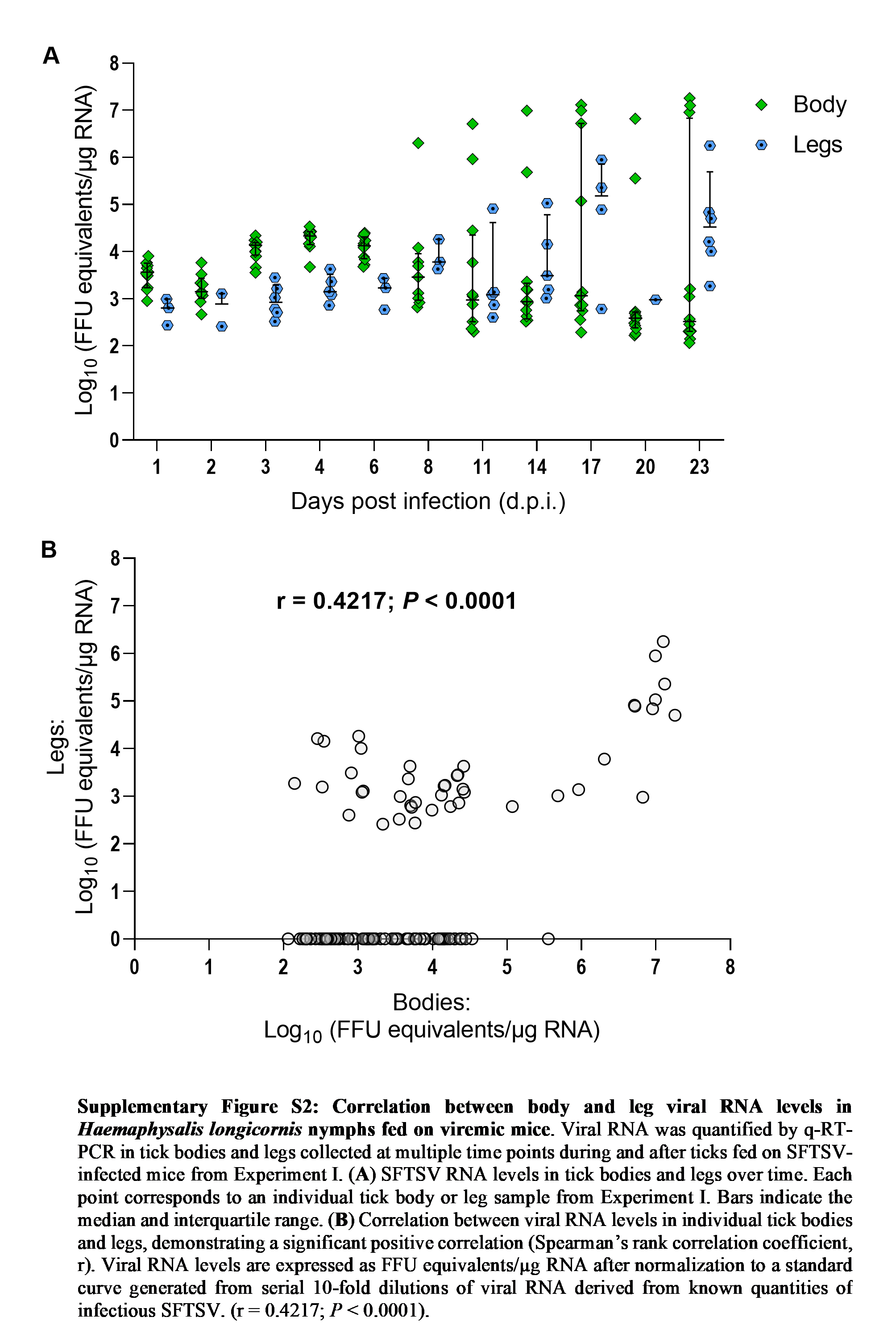

Supplement: Supplementary file 2 [file Image2.tif]
